# Polyphenolic extract of lotus root (edible rhizome of *Nelumbo nucifera*) alleviates hepatic steatosis in obese diabetic *db/db *mice

**DOI:** 10.1186/1476-511X-10-202

**Published:** 2011-11-09

**Authors:** Yumi Tsuruta, Koji Nagao, Shunichi Kai, Keisuke Tsuge, Takashi Yoshimura, Kazuyoshi Koganemaru, Teruyoshi Yanagita

**Affiliations:** 1 Department of Applied Biochemistry and Food Science, Saga University, Saga 840-8502, Japan; 2 Department of Biochemistry and Applied Biosciences, United Graduate School of Agricultural Sciences, Kagoshima University, Kagoshima 890-8580, Japan; 3 Industrial Technology Center of Saga, Saga 849-0932, Japan

## Abstract

**Background:**

Nonalcoholic fatty liver disease (NAFLD) is emerging as the most common liver disease of industrialized countries. Thus, discovering food components that can ameliorate NAFLD is of interest. Lotus root, the edible rhizome of *Nelumbo nucifera*, contains high levels of polyphenolic compounds, and several health-promoting properties of lotus root have been reported. In this study, we tested whether feeding a polyphenolic extract of lotus root to *db/db *mice protects them from hepatic steatosis.

**Results:**

After 3 weeks of feeding, the hepatomegaly and hepatic triglyceride accumulation were markedly alleviated in the lotus polyphenol-diet-fed *db/db *mice relative to the control mice. Although the lipolytic enzyme activity was not changed, the activities of lipogenic enzymes, such as fatty acid synthase and malic enzyme, were significantly lower in the lotus polyphenol diet-fed *db/db *mice. Additionally, the ESI-IT/MS and MALDI-TOF MS spectra revealed the presence of B-type proanthocyanidin polymers with polymerization degree up to 9 in the polyphenolic lotus root extract.

**Conclusion:**

We speculate that the condensed tannins contained in lotus root can alleviate hepatic steatosis by suppressing the lipogenic enzyme activity in the livers of *db/db *mice.

## Background

Nonalcoholic fatty liver disease (NAFLD) is often associated with features of the metabolic syndrome and is emerging as the most common liver disease worldwide [1-4]. NAFLD is the preferred term for describing the spectrum of liver damage that ranges from hepatic steatosis to steatohepatitis, liver fibrosis, and cirrhosis. Most of the liver-related morbidity and mortality are associated with the development of cirrhosis, which is most likely to occur in individuals who have progressed from hepatic steatosis to steatohepatitis. The processes by which steatohepatitis evolves from hepatic steatosis are not fully understood; nevertheless, developing effective therapies for treating NAFLD is necessary, and discovering nutrients that can reduce the risk of NAFLD would be useful. *db/db *mice suffer from hyperphagia, because they have a missense mutation on the leptin receptor gene. They develop a syndrome that involves multiple metabolic and hormonal disorders, including NAFLD, and shares many features with human metabolic syndrome [5-7].

Diet has been recognized as factor that contributes to the development and prevention of NAFLD [8-11], and polyphenol-rich plants and fruits have been used in folk medicines throughout the world for treating lifestyle-related diseases [12-14]. *Nelumbo nucifera *is a plant in the monogeneric family *Nelumbonaceae*. Its rhizome (lotus root) is recognized in eastern countries as one of the most delicious and nutritional vegetables, and it has also been used in traditional Asian herbal medicine. There are several studies showing that lotus root contains high levels of polyphenolic compounds and possesses several beneficial health properties, such as hypoglycemic, anti-inflammatory and antioxidant activities [15-18]. Our previous study showed that feeding lotus root powder prevents the development of NAFLD in obese diabetic *db/db *mice (submitted data). In the present study, we evaluated the effect of a diet supplemented with a polyphenolic extract of lotus root on the development of NAFLD in *db/db *mice. Additionally, the structures of the polyphenolic lotus root extract were characterized through analyses combining a butanolic-HCl assay, electrospray-ionization mass spectrometry (ESI/MS), and matrix-assisted laser-desorption ionization time-of-flight mass spectrometry (MALDI-TOF MS).

## Materials and methods

### Animals and diets

**Figure 1:**
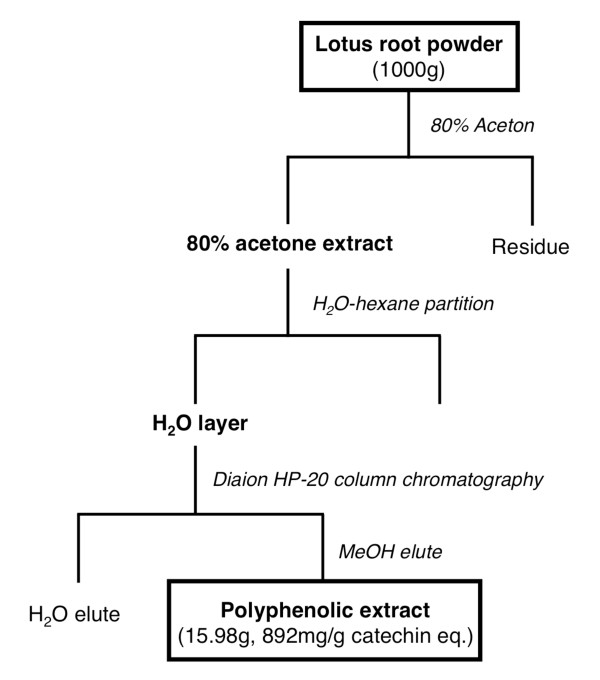
**Preparation of the polyphenolic extracts from the lotus root powder**.

All aspects of the experiment were conducted according to the guidelines provided by the ethical committee for experimental animal care of Saga University. Six-week-old male *db/db *mice (C57BLKS/J Iar-*+Lepr^db^/+Lepr^db^*) were purchased from Japan SLC (Shizuoka, Japan). The mice were housed individually in plastic cages in a temperature-controlled room (24°C) under a 12-h light/dark cycle. The basal semisynthetic diets were prepared according to the recommendations of the AIN-76 [19] (Table 1). The freeze-dried lotus root powder were provided by the Industrial Technology Center of Saga and the preparation of the polyphenolic lotus root extract was performed as shown in Figure 1. Approximately 16 g of polyphenolic extract was obtained from 1000 g of freeze-dried lotus root powder, and the extract included 892 mg/g [(+)-catechin equivalent] total polyphenolic substances, as estimated by the Folin-Ciocalteu method [20]. The *db/db *mice were assigned to two groups (of six mice each) that were fed one of two diets (Table 1): a semisynthetic AIN-76 diet (the control group) or a semisynthetic AIN-76 diet supplemented with 0.5% lotus root polyphenol at the expense of sucrose (the lotus polyphenol group). The mice were pair-fed the diets for 3 weeks using the Rodent CAFE (KBT Oriental Co. Ltd., Saga, Japan). At the end of the feeding period and after a 9-h starvation period, the mice were sacrificed under pentobarbital sodium salt anesthesia by exsanguination from the heart. The white adipose tissue (WAT) and the livers were excised immediately, and the serum was separated from the blood.

**Table 1:** The composition of the experimental diets.

Ingredients	Control	Lotus polyphenol
		**%**
Casein	20.0	20.0
Corn starch	15.0	15.0
Cellulose	5.0	5.0
Mineral mixture (AIN 76)	3.5	3.5
Vitamin mixture (AIN 76)	1.0	1.0
DL-Methionine	0.3	0.3
Choline bitartrate	0.2	0.2
Corn oil	5.0	5.0
Lotus root polyphenol	-	0.5
Sucrose	50.0	49.5

### Measurement of the triglyceride and cholesterol levels in the liver

The liver lipids were extracted according to the method of Folch et al. [21], and the concentrations of triglyceride and cholesterol were measured using the methods of Fletcher [22] and Sperry & Webb [23], respectively.

### Measurement of the serum parameters

The serum triglyceride and cholesterol levels were measured using commercial enzyme assay kits (Wako Pure Chemicals, Tokyo, Japan). The serum glutamic pyruvic transaminase (GPT) and alkaline phosphatase (ALP) activities were measured using commercial enzyme assay kits (Wako Pure Chemicals, Tokyo, Japan).

### Assays of the hepatic enzyme activity

The enzyme activities of fatty acid synthase (FAS) [24] and malic enzyme [25] in the cytosomal fraction and of carnitine palmitoyltransferase (CPT) [26] in the mitochondrial fraction were determined as has been described elsewhere. The protein concentration of each fraction was determined according to the method of Lowry et al. [27], with bovine serum albumin used as the standard.

### Lotus root polyphenol analysis

First, a colorimetric butanolic-HCl assay for procyanidins was employed to assess the polyphenolic lotus root extracts; it used procyanidinB2 as the positive standard and (+)-catechin as the negative standard [28]. Second, a structural analysis of the lotus root polyphenol components was conducted by direct-infusion ESI/MS. The ESI/MS spectrometry was performed using a HCT-Ultra system (Bruker Daltnics GmbH, Bremen, Germany). The sample was dissolved in 50% methanol (1 mg/mL) and directly infused into the ESI/MS system at 0.2 mL/h with a syringe pump. The analytical conditions of the ESI/MS were as follows: ion mode, negative; capillary voltage, 4 kV; capillary exit, -241 V; nebulizing gas, 45 psi; flow rate of dry gas, 10 L/min; dry temperature, 350°C; an scan range, 100-3,000 m/z. Finally, a MALDI-TOF MS analysis was performed to obtain further lotus root polyphenol structural information. The MALDI-TOF MS analysis was conducted using an Autoflex III Smartbeam system (Bruker Daltnics GmbH, Bremen, Germany). The sample was dissolved in 50% methanol (2 mg/mL) and premixed with 2,5-dihydroxybenzoic acid (20 mg/mL in 50% acetonitrile containing 0.1% trifluoroacetic acid) and sodium trifluoroacetate (2 mg/mL in acetonitrile) at a ratio of 2:10:1. Then, 1 μL of the premixed sample solution was deposited onto a stainless steel MALDI target plate and allowed to dry at room temperature. The analytical conditions of the MALDI-TOF MS analysis were as follows: ion mode, reflector positive; ion source 1 voltage, 19 kV; ion source 2 voltage, 16.85 kV; lens voltage, 8.7 kV; paused ion extraction, 100 ns; and mass range, 500-3,000 m/z. The mass spectra were acquired by averaging 1,000 laser shots.

### Statistical analysis

All of the values are expressed as the means ± standard error. The significance of the differences between the means of the two groups was determined by the Student's *t*-test. Differences were considered to be significant at *p *< 0.05.

## Results and Discussion

NAFLD is common in type 2 diabetic and obese patients. Although the mechanisms responsible for the development of NAFLD are unclear, it has been suggested that hepatic steatosis results from increased lipogenesis and/or decreased lipolysis, in addition to the accelerated mobilization of the expanded visceral WAT fat to the liver [2,3].

### Effects of the lotus polyphenol diet on growth parameters in *db/db *mice

**Figure 2:**
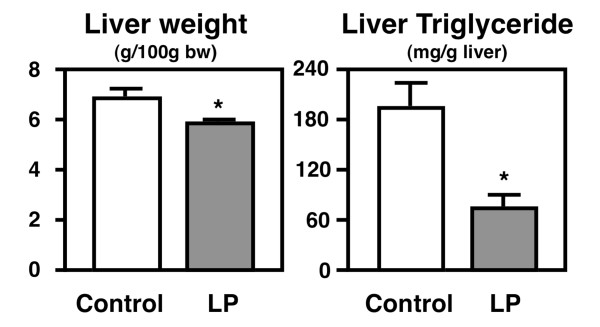
**The relative liver weights and hepatic triglyceride levels in the *db*/*db *mice**. The mice were fed the control diet or the lotus root polyphenol diet for 3 weeks. The values are expressed as the mean ± standard error for six mice. See Table 1 for the composition of the diets. * Significant (*P *< 0.05) difference between the control and lotus polyphenol groups of *db/db *mice.

The two groups of *db/db *mice did not differ in their initial body weight, nor did they differ in their final body weight, food intake, or total WAT weight during and after the 3-week feeding period (Table 2). By contrast, the relative liver weight and hepatic triglyceride concentration differed between the *db/db *mice fed the control and lotus polyphenol diets (Figure 2).

**Table 2:** The effect of polyphenolic lotus root extract on growth parameters in *db/db *mice.

	Control	Lotus polyphenol
Initial body weight (g)	33.7 ± 0.6	33.7 ± 0.5
Final body weight (g)	37.4 ± 0.6	35.8 ± 1.7
Food intake (g)	109 ± 3	109 ± 1
White adipose tissue weight (g/100 g body weight)		
Total	6.92 ± 0.12	6.53 ± 0.25
Epididymal	4.30 ± 0.12	4.10 ± 0.14
Perirenal	2.61 ± 0.06	2.43 ± 0.13

The values are expressed as the mean ± standard error for six mice.

### Effects of Lotus polyphenol diet on hepatic lipids in *db/db *mice

The relative liver weight was 15% less in the lotus polyphenol diet-fed *db/db *mice, and this lower liver weight was associated with markedly less (62%) triglyceride accumulation in the liver (Figure 2). However, the hepatic cholesterol level was not different between the two groups (control group, 2.98 ± 0.19 mg/g liver; lotus polyphenol group, 3.49 ± 0.21 mg/g liver).

### Effects of the lotus polyphenol diet on serum parameters in *db/db *mice

The serum cholesterol levels and triglyceride levels were not different between the two groups (Table 3). Consistent with alleviation of the hepatomegaly and hepatic steatosis by the lotus polyphenol diet, the activities of hepatic injury markers, such as GPT and ALP, tended to be lower (by 24% and 17%, respectively) in the sera of the lotus polyphenol diet-fed *db/db *mice than in the sera of the control diet-fed *db/db *mice (Table 3). These results suggest that the polyphenolic lotus root extract can prevent the development of NAFLD in the *db/db *mice.

**Table 3:** The effect of polyphenolic lotus root extract on serum parameters in *db/db *mice.

	Control	Lotus polyphenol
Triglyceride (mg/dL)	99.8 ± 9.1	106 ± 14
Cholesterol (mg/dL)	166 ± 15	182 ± 10
ALP (IU/L)	40.3 ± 5.5	30.7 ± 2.9
GPT (IU/L)	110 ± 17	91.5 ± 4.6

The values are expressed as the mean ± standard error for six mice.

GPT, glutamic pyruvic transaminase; ALP, alkaline phosphatase.

### Effects of the lotus polyphenol diet on enzyme activity related to lipid metabolism in the livers of *db/db *mice

**Figure 3:**
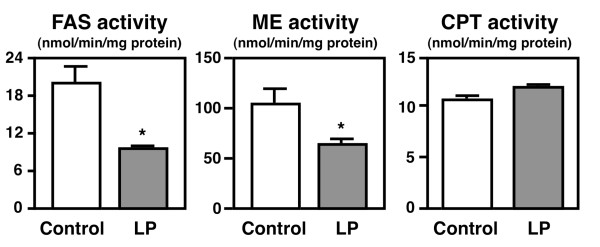
**The activities of the hepatic enzymes related to triglyceride metabolism in the *db*/*db *mice**. The mice were fed the control diet or the lotus root polyphenol diet for 3 weeks. The values are expressed as the mean ± standard error for six mice. See Table 1 for the composition of the diets. * Significant (*P *< 0.05) difference between the control and lotus polyphenol groups of *db/db *mice. FAS, fatty acid synthase; CPT, carnitine palmitoyltransferase; ME, malic enzyme.

To further examine the effect of the lotus polyphenol diet on the liver, the hepatic enzymes related to triglyceride metabolism were analyzed (Figure 3). Although the activity of CPT (a key enzyme in fatty acid β-oxidation) was not changed, the activities of FAS and malic enzyme (lipogenic enzymes related to de novo fatty acid biosynthesis) were significantly lower in the lotus polyphenol diet-fed *db/db *mice. Previous studies have demonstrated that dietary polyphenols suppress lipogenesis. Previous reports have indicated that polyphenols from plants have FAS inhibitory activity *in vitro *[29], and we have reported that feeding tea catechins (which are rich in (-)-epigallocatechin gallate and (-)-epicatechin gallate) to rats results in reduced visceral fat deposition through a reduction of hepatic FAS activity [30]. Given that the lotus root powder in our previous study also suppressed hepatic FAS activity, we speculate that the ameliorative effect of lotus root on hepatic steatosis *in db/db *mice is partly attributable to the suppression of hepatic lipogenic enzyme activity by the lotus root polyphenols.

### Lotus root polyphenol analysis

**Figure 4:**
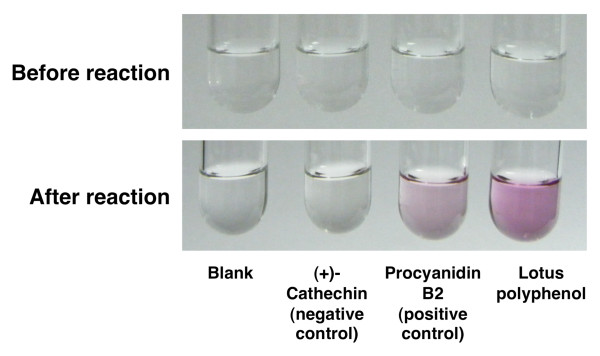
**A qualitative analysis of the lotus root polyphenols by the HCl-butanolic method**. The reaction mixtures consisted of 0.02 mg of (+)-catechin, proanthocyanidin B2, and lotus polyphenols in 500 μl of HCl-butanol (concentrated HCl/*n*-butanol, 1:5) were heated at100°C for 30 min.

**Figure 5:**
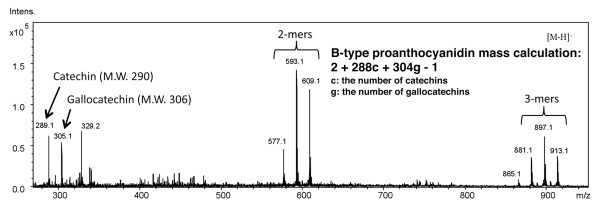
**The negative ion mode ESI/MS spectra of the lotus root polyphenols**.

**Figure 6:**
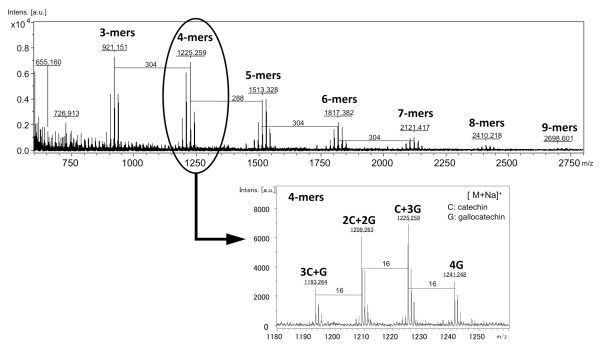
**The positive reflection ion mode MALDI-TOF MS spectra of the lotus root polyphenols**.

**Figure 7:**
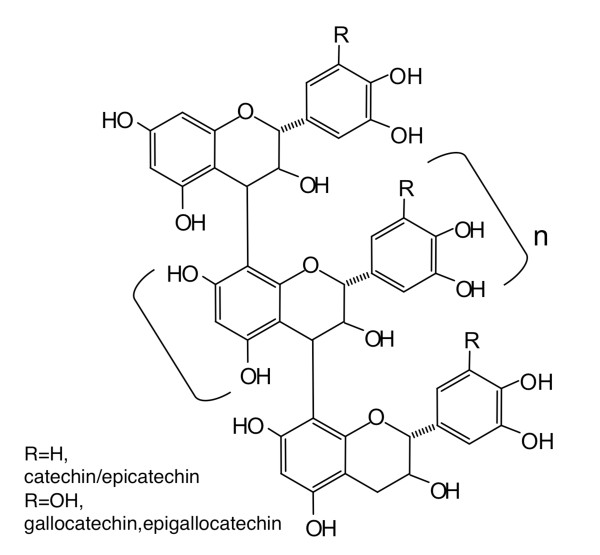
**The putative structure of the condensed tannins in the polyphenolic lotus root extract**.

The colorimetric butanoic-HCl assay showed that the polyphenolic lotus root extract contained proanthocyanidins (Figure 4). The lotus root polyphenols were further characterized by ESI/MS and MALDI-TOF MS spectrometry. The ESI/MS spectra were recorded in the negative ion mode, which detects proanthocyanidin molecules better detected than the positive ion mode, and they showed the existence of epi/catechin, epi/gallocatechin, and B-type proanthocyanidins (Figure 5). The degree of polymerization of proanthocyanidin was calculated by the formula of B-type proanthocyanidin mass calculation. Additionally, both procyanidin (polymeric tannins composed of catechin) and prodelphinidin (polymeric tannins composed of gallocatechin) homopolymers and as a complex series of procyanidin-prodelphinidin heteropolymers with polymerization degree up to 9 were detected through the MALDI-TOF MS analysis (Figure 6). The putative structure of the condensed tannins in the polyphenolic lotus root extract is illustrated in Figure 7. There are several studies reporting that proanthocyanidins possess various *in vivo *physiological properties, such as antioxidant, anti-carcinogenesis, and anti-inflammatory activities [31-33]. Additionally, previous reports have indicated that dietary proanthocyanidins have hypolipidemic properties and that proanthocyanidins from grape seed and persimmon peel down-regulate the expression of lipogenic genes by reducing the steroid response element binding protein 1 transcription factor [34-36]. Deprez *et al*. have shown that proanothocyanidin dimmers and trimmers have similar permeability coefficients in Caco-2 cells [37], and absorption of dimeric and trimeric procyanidins has been demonstrated in rat and human sera [38-40]. Further investigation in future studies will be necessary to evaluate the metabolic fate of lotus root polyphenol and the precise mechanisms of its *in vivo *physiological properties.

## Conclusion

Although further qualitative and quantitative analyses of lotus root polyphenols are necessary, the present results suggest that the ameliorative effect of dietary lotus root on hepatic steatosis in *db/db *mice is attributable to the suppression of hepatic lipogenesis by the lotus root polyphenols.

## List of abbreviations

ALP: alkaline phosphatase; CPT: carnitine palmitoyltransferase; ESI/MS: electrospray-ionization mass spectrometry; FAS: fatty acid synthase; GPT: glutamic pyruvic transaminase; MALDI-TOF MS: matix-assisted laser-desorption ionization time-of-flight mass spectrometry; ME: malic enzyme; WAT: white adipose tissue; NAFLD: nonalcoholic fatty liver disease.

## Competing interests

The authors declare that they have no competing interests.

## Authors' contributions

YT and KN made substantial contributions to the conception and design of the study, to performing the experiment, to the assembly, analysis and interpretation of the data and to drafting the manuscript. SK, KT, and TY participated in the experimental work and in the collection, assembly, analysis of the data. KK and TY contributed to planning the experiment and discussing the results. All authors read and approved the final manuscript.
